# Thermal Death Kinetics of *Cryptolestes pusillus* (Schonherr), *Rhyzopertha dominica* (Fabricius), and *Tribolium confusum* (Jacquelin du Val) Using a Heating Block System

**DOI:** 10.3390/insects10050119

**Published:** 2019-04-26

**Authors:** Lixia Hou, Yi Wu, Shaojin Wang

**Affiliations:** 1 College of Mechanical and Electronic Engineering, Northwest A&F University, Yangling, Shaanxi 712100, China; hlx0924hyx@163.com; 2 Academy of State Administration of Grain, Beijing 100037, China; wuyi@chinagrain.org; 3 Department of Biological Systems Engineering, Washington State University, 213 L.J. Smith Hall, Pullman, WA 99164-6120, USA

**Keywords:** storage insect, thermal treatment, thermal death time model, cumulative lethal time model

## Abstract

Thermal treatment has been extensively used to control pests in stored grains for a long time. The objective of this study was to analyze thermal death kinetics of adult flat grain beetle, *Cryptolestes pusillus* (Schonherr), lesser grain borer, *Rhyzopertha dominica* (Fabricius), and confused flour beetle, *Tribolium confusum* (Jacquelin du Val), using a heating block system (HBS), at temperatures of 46, 48, 50, and 52 °C for *C. pusillus* and *T. confusum*, and 48, 50, 52, and 54 °C for *R. dominica* with a heating rate of 5 °C/min. Thermal death curves of those three insects followed a 0th-order reaction model. Complete mortality of *C. pusillus*, *R. dominica*, and *T. confusum* were observed after exposure to 1.4, 5.0, and 0.9 min at 52, 54 and 52 °C, respectively. The thermal death activation energy for controlling *C. pusillus*, *R. dominica*, and *T. confusum* was 689.91, 380.88, and 617.08 kJ/mol with *z* values of 2.88, 5.18, and 3.22 °C, respectively. The cumulative lethal time model can also be used to predict mortality of these three insects during a practical heating process. The information provided by this study on storage pests may be useful for developing effective thermal treatment protocols.

## 1. Introduction

Flat grain beetle, *Cryptolestes pusillus* (Schonherr), lesser grain borer, *Rhyzopertha dominica* (Fabricius), and confused flour beetle, *Tribolium confusum* (Jacquelin du Val), are major pests of stored products worldwide [1,2,3]. They infest stored grain, beans, nuts, and oil seeds, and could create feeding holes on kernels. Insect infestations of kernels reduce nutritional values, germination rates, and market prices of stored grain, and cause great economic losses to farmers. Therefore, it is necessary to develop effective methods to control these storage pests. Pesticides and fumigants have been widely used to disinfest agricultural products due to their effectiveness and low cost [4]. With increased insect resistance to chemicals and concerns for environmental pollutions, and food safety requirements, there have been renewed efforts to investigate non-chemical insect control methods [5,6].

Thermal treatments are one of the most popular physical treatments to control pests in agricultural products due to their easy applications and non-chemical characteristics [7,8,9]. Before thermal treatment is implemented, the lethal conditions of insects need to be determined because they are changing with different species and life stages [10]. At 45 and 50 °C water bath treatment, 35 and 2 h exposure times were required to control *C. pusillus*, respectively [11]. When wheat was exposed to infrared radiation for 24 h, 99% mortality of *R. dominica* was achieved [12]. Using a grain heating apparatus, LT_99.9_ values of *R. dominica* were between 2.49 h and 78.22 h at treatment temperatures of 53 and 45 °C [13]. After heating for 36 h with 77 electric heaters at 20 kW/h of power, 100% mortality of adult *R. dominica* and *T. confusum* was obtained in a whole flour mill [14]. Such a long heating time required to achieve complete mortality of insects could be caused by slow conventional heat transfer and poor heating uniformity in host materials. Therefore, lethal conditions determined by these methods might be not suitable for rapid microwave and radio frequency (RF) heating. The lethal parameters obtained by the slow or non-uniform heating conditions would result in either insect survivals or product damage in practical thermal treatments. Other researchers reported that holding time for achieving 100% mortality was 1 min at 52 °C for *Plodia interpunctella* [15], 4 min at 50 °C for *Sitophilus oryzae* [16], 4 min at 52 °C for *Sitophilus zeamais* [17], and 1.3 min at 52 °C for *Tribolium castaneum* [18], when heated directly by a heating block system (HBS). These lethal times determined by the HBS were validated using RF treatments. Complete mortality of adult *S. oryzae* infesting milled rice using RF energy with heating rate of 6.1 °C/min was obtained at 50 °C for holding 6 min with hot air [19]. Furthermore, 100% mortality of *T. castaneum* larvae infesting the rapeseeds was achieved with RF treatment at the heating rate of 4.4 °C/min attaining 55 °C without a holding time [20]. The lethal parameters obtained for some insects cannot be applied to other pest spp. without resulting in treatment failures. Therefore, it is necessary to determine the lethal conditions for controlling storage insects, such as *C. pusillus*, *R. dominica*, and *T. confusum*, using RF energy.

Different statistical models have been used to predict the mortality of insects during thermal treatment. Thermal death kinetic (TDK) model is used to successfully predict mortality of insects during isothermal stage when the insects are heated directly by the HBS [17,18]. Cumulative lethal time model calculates the accumulated lethal effect of heating process and holding period by the measured temperature–time history and was used to predict mortality of *Cydia pomonella* in cherries when they were subjected to hot water treatment [21]. Therefore, these two models were used to predict the mortality of insects in this paper.

Since *R. dominica* complete their development inside grain kernels, it is difficult to get internal stages, such as larvae and pupae, for treatments without influencing survival ratio of tested insects. In addition, hand-processing would largely influence survival ratio of eggs and larvae without using host grains for these storage insects. Furthermore, the heating treatments for adult stages in grains are easier to apply, and evaluation of insect survival ratio is immediate and reliable. Therefore, adult stage could be selected to conduct thermal death kinetic studies.

The aims of this study were: (1) to determine thermal survival ratio of adult *C. pusillus*, *R. dominica*, and *T. confusum* at four selected temperatures and different exposure times using the HBS; (2) to develop thermal death kinetic models and calculate activation energy of these three storage insects; (3) to establish mortality curves by a cumulative lethal time model; and (4) to compare the lethal times and kinetic parameters of the three insects to those of other storage pests.

## 2. Materials and Methods

### 2.1. Treatment Procedure

*C. pusillus*, *R. dominica*, and *T. confusum* were reared at the Academy of State Administration of Grain, Beijing, China, and obtained in July 2017. *C. pusillus* and *T. confusum* were reared on 60% wheat flour, 30% oats, and 10% yeast by weight. *R. dominica* was reared on whole wheat (14 ± 1%, w.b.). All the species were contained in glass rearing bottles (9 cm diameter × 10 cm height) sealed with filter papers for air exchange, and maintained at 28 ± 1 °C and 63% RH in a constant temperature and humidity incubator (HWS-350, Hangzhou Aipu Instrument Co., LTD, Hangzhou, China). The adult insects with rearing mixture were separated by a Chinese standard sieve #40 and collected by a glass petri dish (20 cm diameter).

**Figure 1:**
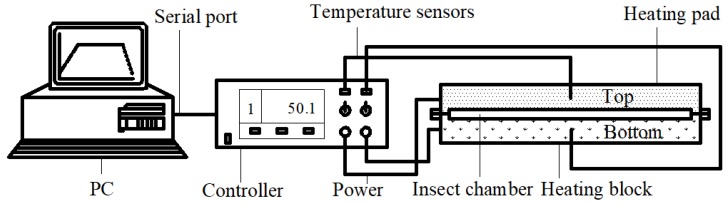
Schematic diagram of heating block system for insect mortality experiments [22].

To obtain survival ratio of different storage insects, the HBS was used, whereby test insects could be heated to target temperatures with heating rates from 0.1 °C/min to 15 °C/min. The system was composed of top and bottom heating blocks, two heating pads, an insect chamber (214 × 214 × 3 mm^3^), temperature sensors, and a data acquisition/control unit (Figure 1). Two block temperatures were measured and collected by two type-T thermocouple sensors (TMQSS-020U-12, Omega Engineering, INC., Norwalk, CT, USA). Heating rate, heating temperature or target temperature, and exposure time, were governed by custom-developed software and proportional-integral-derivative (PID) controllers (i/32 temperature & process controller, Omega Engineering Inc., Norwalk, CT, USA) through a solid-state relay. More information about the HBS can be found elsewhere [22].

Based on lethal conditions of *S. oryzae* [16], *S. zeamais* [17], and *T. castaneum* [18], and preliminary experiments, five exposure times (0.3–150 min for *T. confusum* and 0.2–60 min for *C. pusillus*) at 46, 48, 50, or 52 °C with heating rate of 5 °C/min were chosen to offer a survival ratio from 0% to 100%. For *R. dominica*, five heating times (1–75 min) at 48, 50, 52, and 54 °C, and the heating rate of 5 °C/min were selected. Control insects were put into the unheated HBS for 60 min for *C. pusillus*, 75 min for *R. dominica*, and 150 min for *T. confusum*, which was equivalent to the longest exposure time for each species among the thermal treatments.

To prevent escape of test insects and provide fast extraction from insect chamber, 50 insects were sealed in a nylon-mesh bag (15 × 10 cm) before this was directly placed on the center of the insect chamber. After the bottom heating block was covered with the top one, the treatment program began to run. Once the thermal treatment was finished, test insects were moved to rearing bottles, which were placed under rearing conditions until survival ratio of treated insects was calculated. Since the height of insect chamber was only 3 mm, the heat transfer effect of the nylon-mesh bag on the insect mortality was negligible [16]. Survival ratio of insects was calculated in 48 h after thermal treatment. The insects were considered as dead if the insect was not moving or had no any response to a light probe.

### 2.2. Mortality Models

Survival ratio (%) of treated insects is an important indicator to evaluate effect of thermal treatment and described as the ratio of surviving insects (*N*) to initial numbers of insects (*N*_0_):

(1) $$S=\frac{N}{N_{0}}\times 100\%$$

A fundamental kinetic model, i.e., thermal death time (TDT) kinetic model, was applied to describe the survival ratio of adult *C. pusillus*, *R. dominica*, and *T. confusum* under isothermal treatments and to predict lethal time (LT) at different temperature and survival ratio. According to the TDT kinetic model, the survival ratio of insects and heating time at a certain temperature followed the relationship of Equation (2):

(2) $$\frac{d(N/N_{0})}{dt}=-k({N/N_{0})}^{n}$$

where *t* is the exposure time (min) at certain temperature. *k* means the thermal death rate constant (1/min), and *n* expresses the kinetic order of reactions. Taking the integration of both sides of Equation (2), the survivor ratio as a function of exposure time can be solved with different reaction orders and described as Equation (3):

(3) $$\begin{array}{ll} \ln(N/N_{0})=-kt+c & \left(n=1\right)\\ (N/N_{0}{)}^{1-n}=-kt+c & \left(n\neq 1\right) \end{array}$$

For each temperature, a linear regression was used to analyze survival ratio and exposure time (*t*) on the basis of Equation (3) for the reaction orders of 0, 0.5, 1, 1.5, and 2. The most suitable reaction order was determined by comparing the average coefficients of determination (*R*^2^) over four heating temperatures. Once the reaction order was fixed, the constant values of *k* and *c* were obtained from the regression equation. Then, the best kinetic model was applied to predict the LT_95_, LT_99_, LT_99.33_, LT_99.99_ and 95% confidence interval (CI) for each heating temperature.

TDT curves for adult *C. pusillus*, *R. dominica*, and *T. confusum* were obtained by drawing the minimum exposure time needed to obtain 0% survival ratio of insects at each treated temperature on a semi-logarithmic coordination. The *z* value was calculated as the negative reciprocal of the slope of the TDT curve. Then, the activation energy (*E_a_*, J/mol) was applied to investigate the sensitivity of treated insects to temperature changes and can be estimated by Equation (4):

(4) $$E_{a}=\frac{2.303RT_{\min}T_{\max}}{z}$$

where *R* means the universal gas constant (8.314 J/mol K), *T*_min_ and *T*_max_ are the minimum and maximum temperatures (K) of a test range, respectively. The activation energy for thermal death of treated insects was also deduced from the Arrhenius equation. According to the Arrhenius equation, the activation energy was related to thermal death rate constant (*k*) and absolute temperature (1/*T*) as follows [23]:

(5) $$k=k_{ref}e^{-\frac{E_{a}}{R}(\frac{1}{T}-\frac{1}{T_{ref}})}$$

where *T* is the absolute temperature (K), and *k_ref_* means the thermal death rate constant at the reference temperature (*T_ref_*).

Cumulative lethal time model has been proposed to explore the lethal effect of heating up time and exposure time during the thermal treatment. The cumulative lethal effect of the thermal treatment can be calculated if the actual temperature–time history was recorded during the whole thermal treatment. For any given temperature–time history of *T*(*t*), the cumulative thermal mortality of this thermal treatment can be calculated to an equivalent total lethal time, *M*_ref_ (min), at a reference temperature *T*_ref_ (°C) by using the following equation [23]:

(6) $$M_{ref}=\int_{0}^{t}10^{\frac{T(t)-T_{ref}}{z}}dt$$

where *z* is the temperature difference required for a 10-fold change in the thermal death time curve (°C).

The following relationship between the mortality of treated insects and the cumulated lethal time at the reference temperature of 46 °C was derived from a 0th-order thermal death kinetic model:

(7) $$M\left(\%\right)=1-(-k\times M_{ref}+c)$$

where *M* is the mortality (%) of insects after thermal treatment. *k* and c are constants for the thermal death rate and at the reference temperature, respectively.

### 2.3. Statistical Analysis

Each thermal treatment was repeated three times. The average values and standard deviations were calculated from the three replicates. All statistical analyses were carried out at a 5% significance level using variance and Tukey’s honestly significant difference (HSD) test in the statistical software SPSS 16.0 version (SPSS Inc., Chicago, IL, USA). Statistical analysis of LT was based on non-overlap of 95% confidence intervals (CI). The 95% CI for LT of insects was predicted using commercial statistical software (Minitab 16, Minitab, Shanghai, China).

## 3. Results

### 3.1. Thermal Death Kinetic Model

The survival ratios for control adult *C. pusillus*, *R. dominica*, and *T. confusum*, placed in HBS at 28 °C for 60, 75, and 150 min, were 98.15 ± 0.41%, 98.73 ± 0.52%, and 97.23 ± 0.17%, respectively, indicating that the effect of handling operation on the survival ratio of treated insects was negligible. Accordingly, the survival ratio in temperature–time treatment was not corrected by the high control survival ratio. Table 1 listed coefficients of determination (*R*^2^) for the survival ratio response of *C. pusillus*, *R. dominica*, and *T. confusum* changed with kinetic orders and treated temperatures. Owing to the largest average coefficient of determination (*R*^2^ = 0.97, 0.97, and 0.96) for all treated temperatures, the 0th-order kinetic model was considered as the most suitable one for *C. pusillus*, *R. dominica*, and *T. confusum* and selected for further applications.

**Figure 2:**
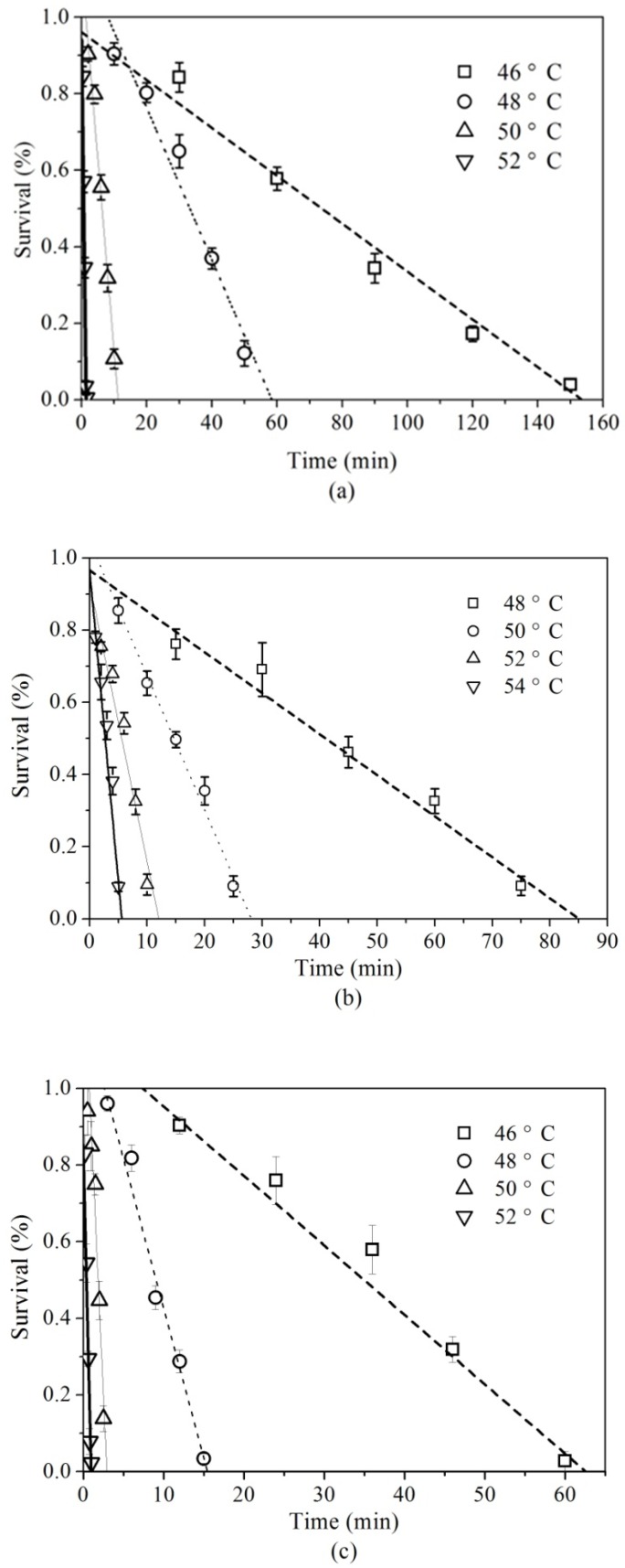
Thermal survival curves at different temperatures and exposure times of adult: *C. pusillus* (**a**); *R. dominica* (**b**); and *T. confusum* (**c**).

The thermal survival curves of adult *C. pusillus*, *R. dominica*, and *T. confusum* for the most suitable 0th-order model are shown in Figure 2. The slopes of the thermal survival curves of these storage insects were reduced sharply when the treated temperature increased from 46 °C to 52 °C for *C. pusillus*, and *T. confusum* and 48 °C to 54 °C for *R. dominica*.

Table 2 lists the model constants obtained from the 0th-order reaction model for thermal survival ratio of *C. pusillus*, *R. dominica*, and *T. confusum*. As expected, the rate constant (*k*) increased with increasing temperature for both conditions, indicating that higher temperatures required shorter exposure time to obtain the same insect mortality. For example, the minimum lethal times to obtain 100% mortality rate of 150 insects for *R. dominica* were about 75.2, 28.7, 11.7, and 5.0 min for 48, 50, 52, and 54 °C, respectively.

Table 3 lists the minimum exposure time for 100% mortality of 150 insects, predicted lethal time (LT) to obtain 95%, 99%, and 99.99% mortality and 95% CI for LT of adult *C. pusillus*, *R. dominica*, and *T. confusum*. Lethal time increased with increasing mortality levels, but decreased with increasing exposure temperatures. When insects heated to 48 °C with the heating rate of 5 °C/min, the values of LT_9__5_, LT_99_, and LT_99.99_ were 73.7, 81.2, and 82.0 min for adult *R. dominica*, respectively. Nevertheless, the values of LT_9__5_ decreased to 4.8 min when the exposure temperature increased to 54 °C. Therefore, a short exposure time at high temperatures dramatically raised the mortality of insects.

**Figure 3:**
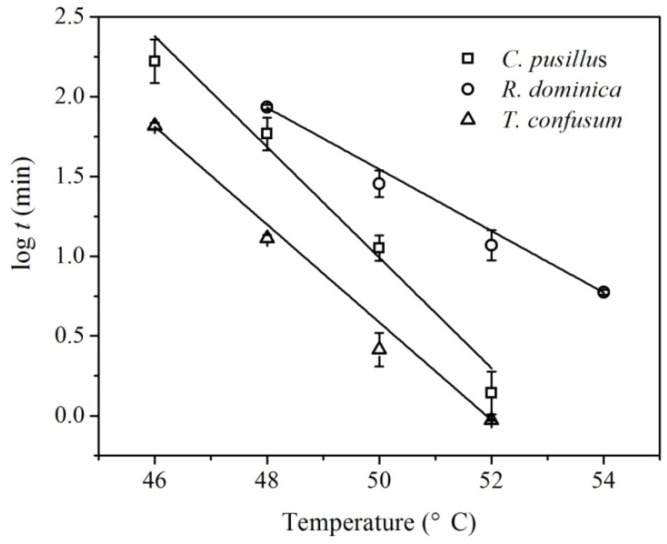
Thermal mortality curve for adult *C. pusillus*, *R. dominica*, and *T. confusum* at heating rate of 5 °C/min.

The TDT curves for adult *C. pusillus*, *R. dominica*, and *T. confusum* are shown in Figure 3. The curves were described by the linear regression equation log *t* = 18.3 − 0.3 × *T* with the coefficient of determination *R*^2^ = 0.98 for *C. pusillus*; log *t* =11.2 − 0.2 × *T* with *R*^2^ = 0.99 for *R. dominica*; and log *t* = 16.1 − 0.3 × *T* with *R*^2^ = 0.99 for *T. confusum*, where *t* is exposure time (min) and *T* means treatment temperature (°C). The *z* values deduced from the negative inverse of the slope of the TDT curve were 2.88 °C, 5.18 °C and 3.22 °C, resulting in thermal death activation energies of 689.91, 380.88 and 617.08 kJ/mol for *C. pusillus*, *R. dominica*, and *T. confusum*, respectively.

**Figure 4:**
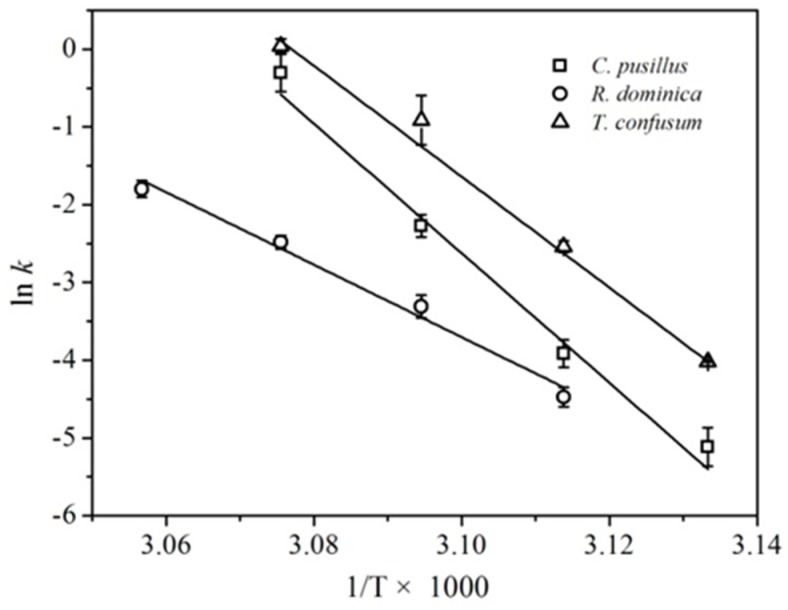
Arrhenius plot for temperature effects on thermal death rates of adult *C. pusillus*, *R. dominica*, and *T. confusum.*

Figure 4 lists the Arrhenius graph for temperature influences on thermal death rates of adult *C. pusillus*, *R. dominica*, and *T. confusum*. The regression equations were ln *k* = 255.8 − 83.4 × 1/*(T* × 1000) for *C. pusillus*, ln *k* = 140.6 − 46.6 × 1/(T × 1000) for *R. dominica*, and ln *k* = 220.4 − 71.6 × 1/(T × 1000) for *T. confusum*. From the slope of the regression equation, the activation energy obtained by Equation (5) for *C. pusillus*, *R. dominica*, and *T. confusum* was 692.97, 387.07, and 595.45 kJ/mol, respectively, which agreed with that calculated by Equation (4). The activation energy of *T. confusum* was smaller than that for *C. pusillus*, suggesting that *T. confusum* was more sensitive to temperature changes than *C. pusillus*. Although *R. dominica* had smaller activation energy, it was more heat resistant than *C. pusillus* and *T. confusum*, since *R. dominica* was treated in a higher temperature range.

### 3.2. Cumulative Lethal Time Model

**Figure 5:**
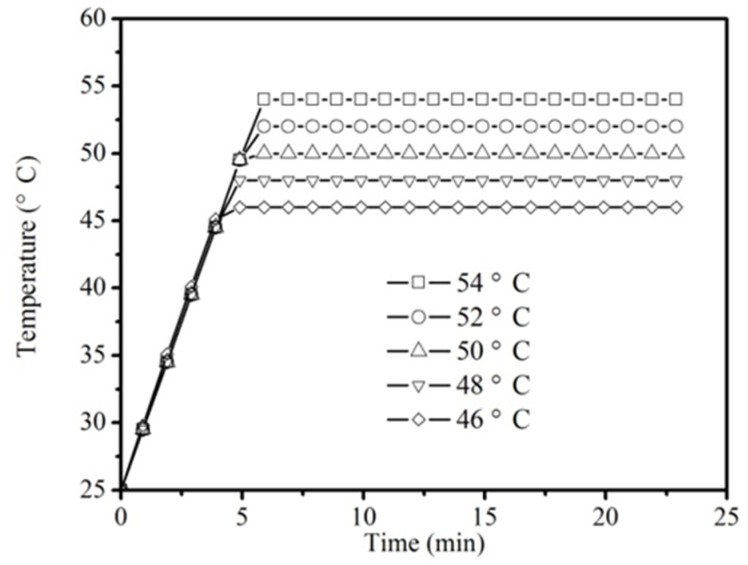
Temperature–time histories for the thermal treatment.

Figure 5 shows an actual temperature–time history of top and bottom blocks during thermal treatment. The temperatures of top and bottom blocks increased at a heating rate of 5 °C/min during ramp period. Once reaching the target temperatures, the temperatures of top and bottom blocks became constant during the holding period. The heating up times were 3.6, 4.0, 4.4, 4.8, and 5.2 min at target temperatures of 46, 48, 50, 52, and 54 °C, respectively.

**Figure 6:**
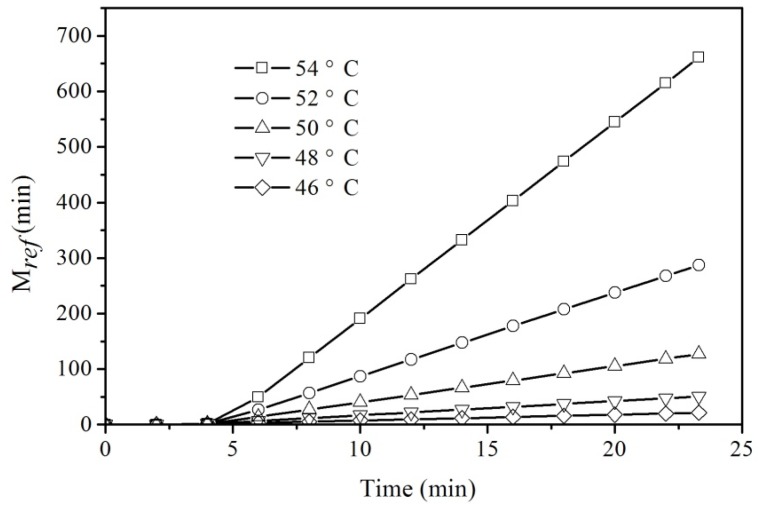
The cumulative lethal time *M_ref_* for *R. dominica* as a function of treatment time at four heating temperatures at the reference temperature of 46 °C.

From the temperature–time curve, the cumulative lethal time *M_ref_* calculated from Equation (6) is shown in Figure 6. The cumulative lethal time increased slowly during ramp periods, and then increased linearly and quickly during the holding period. For example, the cumulative lethal times were 0.2, 49.5, 155.6, and 261.9 min when the heating times were 3, 6, 9, and 12 min at 54 °C, respectively. The cumulative lethal times *M_ref_* at the end of ramp periods were 0.5, 1.2, 3.0, 7.2, and 17.6 min at 46, 48, 50, 52, and 54 °C when the reference temperature was 46 °C, respectively. According to Equation (6), the cumulative lethal times *M_ref_* for *T. confusum* were 63.7, 66.2, 62.4, and 65.7 min when the minimum lethal times were 62.4, 12.9, 2.6, and 0.9 min at 46, 48, 50, and 52 °C, respectively. Furthermore, the average cumulative lethal times *M_ref_* for *C. pusillus*, *R. dominica*, and *T. confusum* were 155.4 ± 4.7, 179.9 ± 6.3, and 64.5 ± 1.8 min at 46 °C, respectively, indicating that the thermo-tolerance of these three storage insects after exposure to thermal treatment was in the following order: *R. dominica* > *C. pusillus* > *T. confusum*.

The results indicate that cumulative lethal time model could be used for predicting the mortality of insects with the help of thermal death kinetics model and the error value was less than 5%. On the other hand, the cumulative lethal times *M_ref_* of heating up time could be calculated based on different *z* values and were 0.327 ± 0.004, 0.501 ± 0.002, and 0.332 ± 0.004 min for *C. pusillus*, *R. dominica*, and *T. confusum*, respectively. The minimum lethal times were several minutes at high temperatures or dozens of minutes at low temperatures, indicating that the effect of heat accumulation during ramp period was little for the whole lethal treatment. Moreover, the cumulative lethal times *M_ref_* of heating up time were almost constant for treated insects in this study. That is, the thermal death kinetic model can be used to predict the mortality of insects with considering the ramp period based on *z* value for different insects.

## 4. Discussion

Survival ratios of adult *C. pusillus*, *R. dominica*, and *T. confusum* followed 0th-order kinetic reaction models. This order reaction model corresponds to that for *Conogethes punctiferalis* [24], *S. oryzae* [16], and *S. zeamais* [17], but contrasts with other studies using 0.5th-order reaction model [15,18,25,26]. Lethal time of treated insects increased with increasing mortality levels, but decreased with increasing exposure temperatures. Similar results were found on *C. pusillus* and *T. confusum*. Exposure times for *C. pusillus* to obtain 95% mortality were 158.0 min at 46 °C but 1.3 min at 52 °C. This may be caused by water loss and denaturing enzyme in insects at high temperatures. When the heating temperature was higher than the threshold value, cuticular permeability and water loss of insect body increased [27]. Then, ionic concentration in body fluids increased with increasing heating temperature, which accelerated the damage of cells. Pyruvate kinase, a key enzyme in glycolysis, was denatured at higher temperatures, which resulted in metabolic disorder and insect body damage or even death [10,28,29]. The minimum exposure time for 100% mortality of *R. dominica* reported by others was longer than observed one in this study since they heated insects with culture substrate or stored grain [13,30]. Due to the long minimum lethal time compared to *C. pusillus* and *T. confusum* at same treated temperatures, *R. dominica* was more thermal resistant than *C. pusillus* and *T. confusum*. Since *R. dominica* had higher *z* value than *C. pusillus* and *T. confusum*, it was more heat resistant than *C. pusillus* and *T. confusum*. This result revealed by *z* value was consistent with that indicated by the minimum exposure time.

Table 4 shows the comparison of lethal times and kinetic parameters of different storage pests reported in the literature. Most of stored pests followed the 0th-order reaction except for *P. interpunctella* and *T. castaneum*, which followed the 0.5th-order reaction. The *k* value of storage pests increased with increasing temperature, indicating that higher temperatures required shorter exposure time to obtain the same insect mortality. This was validated by the minimum heating time for 100% insect mortality, since it needed 61, 14, 3, and 1 min for *T. confusum* to reach 100% mortality at 46, 48, 50, and 52 °C, respectively. In general, lethal temperature and exposure time of insects depended on species, developmental stage, acclimation temperature, acclimation time, heating rate, and relative humidity [10]. In Table 4, it can be concluded that *R. dominica* is the most thermal resistant insect among the storage pests investigated because the longest lethal time (5 min) was needed at 54 °C to achieve 100% mortality. Due to industry-scale treatment needs high throughputs, an effective thermal treatment protocol by combining 55–60 °C with holding several minutes or higher treated temperature without holding could be developed to control insects in grains or other agriculture products.

## 5. Conclusions

Survival ratios of adult *C. pusillus*, *R. dominica*, and *T. confusum* were influenced by different thermal treatments, including target temperature and exposure time. Survival ratios of these three storage insects followed a 0th-order kinetic reaction model. The minimum exposure times needed for achieving 100% mortality of *C. pusillus*, *R. dominica*, and *T. confusum* were 1.4, 5.0, and 0.9 min at 52, 54, and 52 °C, respectively. The activation energy for controlling adult *C. pusillus*, *R. dominica*, and *T. confusum* was 689.91, 380.88, and 617.08 kJ/mol while the *z* value obtained from the TDT curve was 2.88, 5.18, and 3.22 °C, respectively. The thermal resistance of *R. dominica* was higher than that of *C. pusillus*, followed by that of *T. confusum*. The mortality of storage insects could be predicted by the cumulative lethal time model based on the temperature–time history during the heating process. The results of this study may provide useful information for controlling insects in stored grains and cereals.

## Figures and Tables

**Table 1:** Determination of the suitable kinetic order (*n*) for the thermal death of *C. pusillus*, *R. dominica*, and *T. confusum* at four temperatures by comparing the coefficients of determination (*R*^2^).

Temp. (°C)	*N*_0_	*n* = 0	*n* = 0.5	*n* = 1.0	*n* = 1.5	*n* = 2.0
*C. pusillus*						
46	150	0.98	0.99	0.92	0.79	0.66
48	150	0.97	0.92	0.85	0.76	0.68
50	150	0.99	0.96	0.89	0.80	0.71
52	150	0.96	0.96	0.83	0.72	0.65
Average	150	0.97	0.96	0.87	0.87	0.67
*R. dominica*						
48	150	0.98	0.96	0.88	0.77	0.67
50	150	0.98	0.93	0.83	0.73	0.64
52	150	0.96	0.91	0.83	0.74	0.66
54	150	0.97	0.88	0.98	0.97	0.95
Average	150	0.97	0.92	0.88	0.80	0.73
*T. confusum*						
46	150	0.98	0.91	0.78	0.65	0.58
48	150	0.98	0.95	0.82	0.67	0.57
50	150	0.93	0.87	0.80	0.84	0.65
52	150	0.96	0.99	0.99	0.72	0.75
Average	150	0.96	0.93	0.72	0.75	0.64

*N*_0_, the initial number of insects tested.

**Table 2:** Thermal death constants of 0th-order reaction model for *C. pusillus*, *R. dominica*, and *T. confusum* at four different temperatures.

Temp. (°C)	(*N/N*_0_)^1−0^ = −*k t* + *c*	
	*k*	*c*
*C. pusillus*		
46	0.01	0.99
48	0.02	1.17
50	0.10	1.16
52	0.74	1.03
*R. dominica*		
48	0.01	0.98
50	0.04	1.03
52	0.08	0.98
54	0.20	0.99
*T. confusum*		
46	0.02	1.18
48	0.08	1.03
50	0.40	1.03
52	1.04	0.98

**Table 3:** Comparison of LTs (min) obtained by experiments and predicted by 0th-order kinetic models for adult *C. pusillus*, *R. dominica*, and *T. confusum* at four different temperatures.

Temp. (°C)	*N*_0_	Min. Exposure Time for 100% Mortality of 150 Insects	Predicted Treatment Time (min) (95% CI)		
			LT_95_	LT_99_	LT_99.99_
*C. pusillus*					
46	150	153.3	158.0 (123.9–157.2)	164.7 (128.6–164.3)	166.3 (129.7–166.0))
48	150	58.5	59.9 (45.2–65.1)	61.9 (46.6–67.7)	62.4 (46.9–68.3)
50	150	11.1	11.3 (9.5–11.8)	11.7 (9.8–12.2)	11.8 (9.8–12.4)
52	150	1.4	1.3 (1.1–1.5)	1.4 (1.1–1.5)	1.4 (1.1–1.6)
*R. dominica*					
48	150	75.2	73.7 (68.8–92.7)	81.2 (71.4–96.9)	82.0 (72.1–98.3)
50	150	28.7	29.9 (24.2–29.6)	30.4 (25.1–30.9)	28.6 (25.3–31.2)
52	150	11.7	11.9 (8.9–13.0)	12.4 (9.2–13.6)	12.5 (9.2–13.8)
54	150	5.0	4.8 (4.4–6.6)	4.9 (4.6–6.9)	5.0 (4.6–7.0)
*T. confusum*					
46	150	62.4	64.9 (54.5–68.1)	67.1 (56.1–70.6)	67.9 (56.6–71.3)
48	150	12.9	13.5 (13.1–16.3)	14.4 (13.5–16.9)	14.7 (13.6–17.1)
50	150	2.6	2.9 (2.1–3.6)	3.0 (2.1–3.7)	3.1 (2.1–3.8)
52	150	0.9	0.9 (0.7–1.0)	0.9 (0.8–1.1)	0.9 (0.8–1.1)

*N*_0_, the initial number of insects tested.

**Table 4:** Comparison of lethal times and kinetic parameters for thermal treatment between adult lesser grain borer and other storage pests.

Insect	Temp. (°C)	Min. Time for 100% Mortality (min)	Thermal Death Constant		Reaction Order	*Z* (°C)	Activation Energy (kJ/mol)	Source
			*k*	*c*				
*Cryptolestes pusillus*	46	153	0.0059	1.1100	0	2.88	689	This study
	48	58	0.0149	1.1692				
	50	11	0.0788	1.1692				
	52	1	0.8192	1.2601				
*Plodia interpunctella*	44	120	0.0078	1.069	0.5	3.9	514	[15]
	46	30	0.0313	0.963				
	48	10	0.0999	0.983				
	50	3	0.3595	1.011				
*Rhyzopertha dominica*	48	75	0.0114	0.9784	0	5.2	381	This study
	50	29	0.0366	1.0387				
	52	12	0.0837	0.9813				
	54	5	0.1657	0.9862				
*Sitophilus oryzae*	44	130	0.0069	1.0485	0	3.9	505	[16]
	46	50	0.0148	0.9913				
	48	12	0.0650	1.0235				
	50	4	0.2499	1.0560				
*Sitophilus zeamais*	46	160	0.0061	1.0100	0	3.8	527	[17]
	48	40	0.0244	1.0360				
	50	14	0.0635	0.9424				
	52	4	0.2495	0.9616				
*Tribolium castaneum*	48	85	0.0122	1.0365	0.5	2.5	814	[18]
	50	12	0.1042	1.0509				
	52	2	0.5182	0.9075				
*Tribolium confusum*	46	62	0.0160	1.2317	0	3.22	616	This study
	48	13	0.2898	1.1940				
	50	3	0.0654	1.2438				
	52	1	1.0118	1.1361				
